# PHARYNGEAL ELECTRICAL STIMULATION TO TREAT DYSPHAGIA IN ACUTE STROKE: LEARNINGS FROM CASES IN THE PHEED CLINICAL TRIAL

**DOI:** 10.2340/jrm.v57.43538

**Published:** 2025-09-05

**Authors:** Richard L. HARVEY, Richard SMITH, Rajaram BATHULA, Lisa EVERTON, Nicole RUP, Jeff SAVER, Bonnie MARTIN-HARRIS, Rainer DZIEWAS, Satish MISTRY, Shaheen HAMDY, Philip M. BATH

**Affiliations:** 1 Brain Innovation Center, Shirley Ryan Ability Lab, Chicago, IL, USA; 2 Stroke Neurorehabilitation, St Anthony Hospital, CommonSpirit, Lakewood, CO, USA; 3 Stroke, London Northwest University Healthcare NHS Trust, Northwick Park Hospital, Harrow, UK; 4 Adult Speech & Language Therapy Department, Nottingham University Hospitals NHS Trust, Nottingham, UK; 5 Carolinas Rehabilitation NorthEast, Atrium Health, Concord, NC, USA; 6 Department of Neurology, David Geffen School of Medicine at UCLA, Los Angeles, CA, USA; 7 School of Communication, Feinberg School of Medicine, Northwestern University, Evanston, IL, USA; 8 Department of Neurology and Neurorehabilitation, Klinikum Osnabrueck, Osnabrueck, Germany; 9 Phagenesis Ltd, Manchester, UK; 10 Division of Diabetes, Endocrinology and Gastroenterology, School of Medical Sciences, University of Manchester, Manchester, UK; 11 Stroke, Nottingham University Hospitals NHS Trust, Nottingham, UK; 12 Stroke Trials Unit, Mental Health & Clinical Neuroscience, University of Nottingham, Nottingham, UK

**Keywords:** deglutition disorders, electrical stimulation therapy, pharynx, stroke

## Abstract

**Objective:**

To assess the efficacy of pharyngeal electrical stimulation in improving dysphagia post-stroke.

**Design:**

A randomized, sham-controlled, blinded multicentre clinical trial.

**Subjects/Patients:**

Seventeen patients with acute ischaemic or haemorrhagic stroke experiencing dysphagia, indicated by a penetration aspiration scale score of 4–8 on videofluoroscopy.

**Methods:**

Sites enrolled 3 open-label roll-in participants and then randomized subsequent participants to either stimulation or sham treatment. Study interventions were delivered for 10 min daily over 3 consecutive days. Prior to data lock the primary outcome was modified to the change in dysphagia severity rating scale from pre-treatment to end of follow-up period. Secondary outcomes included penetration-aspiration scale score assessed via videofluoroscopy 48 h after final treatment and functional oral intake scale, measured at 7, 14, and 83 days post-randomization.

**Results:**

The trial was halted early due to low recruitment, with 15 participants receiving active stimulation and 2 receiving sham treatment. Active stimulation significantly reduced dysphagia severity at day 83 (difference: –4, *p* = 0.027). Improvements were observed in diet and supervision subscales, and functional oral intake scores. Of those treated, 67% were discharged home, with no serious adverse events attributable to the intervention noted in either group.

**Conclusion:**

Pharyngeal electrical stimulation was safe and associated with reduced dysphagia severity in stroke patients, warranting further validation in larger studies.

## LAY ABSTRACT

This study aimed to evaluate the effectiveness of a new treatment, pharyngeal electrical stimulation, for patients who have difficulty swallowing (dysphagia) after a stroke. Researchers conducted a clinical trial with 17 patients who had recently suffered a stroke and experienced swallowing issues. Participants were randomly assigned to receive either the electrical stimulation or a placebo treatment for 3 days. Although the trial was stopped early because of low identification of appropriate study participants, results showed that those who received the actual treatment had a significant improvement in their swallowing abilities after 83 days. Specifically, 67% of these patients were able to return home after treatment, and no serious side effects were reported. Overall, the findings suggest that pharyngeal electrical stimulation is a safe option that may help improve swallowing difficulties in stroke patients, indicating the need for further research with larger groups.

Dysphagia is common following acute stroke with an incidence of up to 78% (1–4). The majority of stroke survivors recover swallowing function, but 11–40% with more severe stroke impairment remain with dysphagia beyond 6 months (4, 5). The presence of dysphagia poses a high risk for aspiration pneumonia (~25%) and a nearly 6-fold increase in the risk of death in the acute care setting (1, 2, 4, 6, 7). Further, post-stroke dysphagia (PSD) is associated with the need for intubation and ventilation in some patients, and many patients develop malnutrition, require enteral feeding, have poor functional outcome and quality of life, longer institutionalization, and higher financial costs of care (1, 4, 7–9).

Conventional management of dysphagia includes behavioural interventions such as swallowing exercises and altered dietary consistencies aimed to reduce aspiration, and compensatory postures or swallowing manoeuvres as well as frequent oral hygiene are often recommended (10). A variety of interventions have been tested for the treatment of PSD (11, 12). One of these, pharyngeal electrical stimulation (PES), is a novel and innovative neurostimulation treatment for restoring the neurological control of swallowing in patients with dysphagia that has been commercially available following Conformité Européenne (CE) certification in Europe in 2012, and more recently was approved by the United States Food & Drug Administration (FDA). Moreover, there is a growing body of literature suggesting that PES is effective in reducing PSD (13). Among patients with neurogenic dysphagia from various types of acquired brain injuries, PES is associated with improved airway protection, reduced aspiration, and decannulation of tracheostomized patients within 24–72 h of treatment and improved feeding status, consequently resulting in shorter hospital stays with no serious adverse events (14–24). As a result, PES has been included in guidelines for the treatment of neurogenic dysphagia and tracheostomy care, and in the management of swallowing disorders in ICU patients (25–28).

The present study was designed as a pivotal trial with a primary objective to determine whether PES is effective in reducing the severity of unsafe swallows in the treatment of acute dysphagia following hemispheric stroke as a part of the application to secure US FDA approval for PES. Our hypothesis was that PES-treated patients would have reduced penetration and aspiration events on videofluoroscopy study (VFS) as compared with patients given sham treatment. We also hypothesized that PES would reduce swallowing impairment and improve tolerance of less restrictive food and liquid consistencies.

## METHODS

### Study design

PhEED (Pharyngeal Electrical stimulation to trEat Dysphagia) was an international prospective, randomized, sham-controlled, patient-masked, outcome assessor-blinded, multicentre study, which had an adaptive group sequential design with unblinded sample size re-estimation. The study clinical investigation plan (CIP) is available at: https://clinicaltrials.gov/study/NCT03358810. This report follows the CONSORT guidelines for randomized controlled trials.

### Setting

Recruitment and follow-up took place between July 2018 and January 2020 at 11 secondary/tertiary care centres in the USA, UK, and Germany. Analyses were completed in March 2021.

### Study population

Patients were eligible for the study if they were adults (age 18–90 inclusive), were 7–28 days after ischaemic or haemorrhagic stroke, were conscious (NIHSS level of consciousness score 0 or 1), had moderate–severe dysphagia with a penetration aspiration scale (PAS) score of 4–8 on VFS in at least 3 thin liquid boluses and were willing to have a PES catheter inserted. Key exclusion criteria were severe stroke (National Institutes of stroke scale, NIHSS score > = 25), inability to communicate due to severe dysphasia (NIHSS best language score 3), dysphagia due to other causes (e.g., traumatic brain injury, subarachnoid haemorrhage, multiple sclerosis, tumour, dementia), presence of an implanted cardiac pacemaker or cardioverter defibrillator, pregnancy, or a nursing mother. Full inclusion and exclusion criteria are listed in the Clinical Investigation Plan (CIP, available at https://clinicaltrials.gov/study/NCT03358810).

### Approvals and training

The study was funded and sponsored by the manufacturer, Phagenesis Ltd (Manchester, UK), and approved by Institutional Review Boards and Research Ethics Committees. Participants signed a standard approved informed consent form explaining the study and conditions for participation. Proxy consent from a legal authorized representative was permitted if a patient lacked capacity and this was allowed in the relevant jurisdiction. All sites received face-to-face training in the study CIP and delivery of PES provided by the manufacturer. The trial was registered at https://clinicaltrials.gov/study/NCT03358810.

### Randomization

Each site initially recruited 3 open-label roll-in PES-treated participants. The site’s subsequent patients were then randomized to either PES or sham PES. PES was delivered for 10 min on 3 consecutive days. Randomization was stratified by site and baseline PAS and minimized with probabilistic allocation based on key prognostic factors including baseline PAS, age, and NIHSS.

### Intervention

The device used to deliver PES was the CE-marked Phagenyx^®^ Neurostimulation System (Phagenesis Ltd, Manchester, UK); the CE mark covers the treatment of neurogenic dysphagia and devices were used as marketed in the European Union (EU). Within the EU the device was not considered investigational; however, in the United States this device was at the time of the trial marked as an investigational device with an investigational device exemption. The treatment catheter is a specially designed single-patient-use device with built-in stimulation electrodes that doubles as a feeding tube if required. Pharyngeal stimulation was provided at 5 Hz for 10 min on each of 3 consecutive days (1 cycle) (14–17). The stimulation intensity was set at 75% of the maximal tolerable intensity (or first point of discomfort) above the perceptual threshold of stimulation, and was calibrated before commencement of PES on each treatment day. Subjects assigned to the control group received 10-min sham treatments on 3 consecutive days using a Base Station that did not deliver any electrical current, including during simulated identification of threshold and tolerance levels using the sham base station. All subjects were told that they may or may not feel the stimulation to maintain blinding.

### Outcome

The primary outcome measure for the planned clinical trial was the validated 8-level PAS score of each swallow (29) measured instrumentally using VFS at baseline and 48 h following the last investigational treatment. PAS was scored by trained study speech-language pathologists. Each participant provided up to 12 repeated swallow measurements including 6 boluses of 5 mL thin (Bracco Varibar^®^ thin liquid barium sulphate suspension, 40% w/v after reconstitution, target viscosity 4 centipoise, cps; Bracco Diagnostics, Monroe, NJ, USA) and 6 boluses of 5 mL nectar (Varibar^®^ nectar barium suphate oral suspension, 40% w/v, target viscosity 300 cps). The thin and nectar consistencies map to the international dysphagia diet standardization initiative (IDDSI) (30) for levels 0 and 2 respectively. VFS was collected using continuous fluoroscopy or at 25–30 pulses/s with most sites using a 2000 SP TIMS DICOM System (Foresight Imaging, Chelmsford, MA, USA). Further discussion of the primary outcome and its modification is discussed in the changes to the statistical analysis plan.

Secondary outcomes at days 7, 14, and 83 after randomization included the validated 15-level dysphagia severity rating scale (DSRS) (17, 31) and 7-level functional oral intake scale (FOIS) (32). The DSRS has 3 domains (diet, fluid, and supervision), each scored 0 to 4. A score of 0 on each domain indicated normal food texture, thin liquid, and feeding independence and scores of 4 indicated inability to tolerate any liquid or food (see Table IV). The FOIS has a minimal score of 1 indicating nothing by mouth to 7 indicating unrestricted diet. Other outcomes included: quality of life using the EQ-5D; time to transition from enteral feeding (i.e., removal of NG tube or PEG, transition to oral feeding, or first diet upgrade); time to discharge from site; discharge destination (home or institution); days on antibiotics during hospital stay; stroke impairment (NIHSS), disability using Barthel index (BI), function with modified Rankin scale (mRS); new-onset pneumonia as defined by Kalra and colleagues (33); hospital readmission rate; and number of chest X-rays (for suspected pneumonia). Treatment optimization parameters (threshold, tolerance, and stimulation intensity) were recorded on each of the 3 treatment days for subjects who received active treatment. Vital sign monitoring (heart rate, blood pressure, and 30-s ECG rhythm strips) were collected in all patients before, during, and post-treatment. Serious adverse events (SAEs), serious adverse device effects (SADEs), and deaths were recorded at day 83.

Additionally, modified barium swallow impairment profile (MBSImP) metrics (overall impression scores) (34, 35) were determined by the core lab (Swallowing Cross-Systems Collaborative at Northwestern University, Director BM-H) through physiological measurements of each thin and nectar liquid bolus.

### Statistical analysis

Sample size and power were determined through simulations. In order to achieve 180 evaluable patients with 7-day (+/- 1 day) data and assuming a 20% dropout rate, approximately 225 patients were planned for enrolment initially. An interim analysis for futility (non-binding stop) was to occur after the first 60 patients completed their day 7 visit, and another interim analysis was to be performed for efficacy after 120 patients for futility stopping, early efficacy stopping on the primary endpoint and secondary endpoint, and sample size reassessment (SSR). The total sample size could be increased up to approximately 338 patients after the second interim analysis to ensure up to 270 evaluable patients.

Based on a prior feasibility study (14), the primary endpoint was hypothesized to have an odds ratio of 2.23 favouring the PES group. For the secondary endpoints, the difference in mean DSRS between treatment groups was expected to be 1.75 with standard deviation of 4 in both treatment groups. With these effect sizes, the power is 95% for significance on the primary endpoint and 85% for significance on both endpoints.

The primary efficacy analysis was based on an ordinal logistic model of PAS (1–8 scale) of each bolus, determined by VFSS 48 h following the last investigational treatment, and converted to a trichotomized response of safe (PAS 1–3), penetration (PAS 4–5), or aspiration (PAS 6–8). A superiority test was planned on the main effect of treatment as the difference in cumulative log odds between treatment groups. If the primary analysis was positive then secondary endpoints would be analysed: DSRS using ANCOVA adjusted for baseline; and FOIS using the Cochran–Mantel–Haenszel (CMH), stratified by site and baseline PAS, and using the modified ridit score.

No imputation was to be performed for missing data, and no adjustment was made for multiplicity of testing. *P* < 0·05 is considered significant. These approaches were formulated in a statistical analysis plan (dated 1 February 2019): https://clinicaltrials.gov/study/NCT03358810.

### Changes to statistical analysis plan

Trial recruitment was significantly lower than planned and the trial was stopped early with 14 participants treated with open-label PES and 3 randomized, 1 to PES and 2 to sham. As a result, it would not be possible to compare PES with sham as originally planned. Further, incomplete data were present for VFS-assessed PAS. Hence, prior to data lock, a decision was made to revise the analyses to follow the existing published approach used in the PHADER phase IV open-label single-arm study (15), focusing on DSRS as the primary outcome and comparing results at end of follow-up with pre-treatment values (before–after comparisons). All decisions on analysis were made prior to data lock and data visualization. Patients who died were not included in analyses, including for scales that intrinsically document death (e.g., mRS, EQ-5D). Non-parametric descriptors (median [interquartile range]) and tests were used due to the small data size. Where possible, the 1-sample exact permutation test was used to calculate the median difference (MD) and 95% confidence intervals (95% CI) for paired before–after data. Otherwise, the Mann–Whitney *U* test was used to estimate difference in medians (DIM) and 95% CI for unpaired before–after data.

### Role of the study sponsor

The study sponsor was involved in the design and conduct of the study, data management, compensated sites for data collection, and reviewed and approved the manuscript. The initial statistical analysis plan was prepared by Cytel Inc (Cambridge, MA, USA). The decision to revise the analysis plan to follow that used in the PHADER study (15) (where possible) was made by PMB. All authors had full access to all data. The corresponding author had final responsibility for the decision to submit for publication.

## RESULTS

**Fig. 1:**
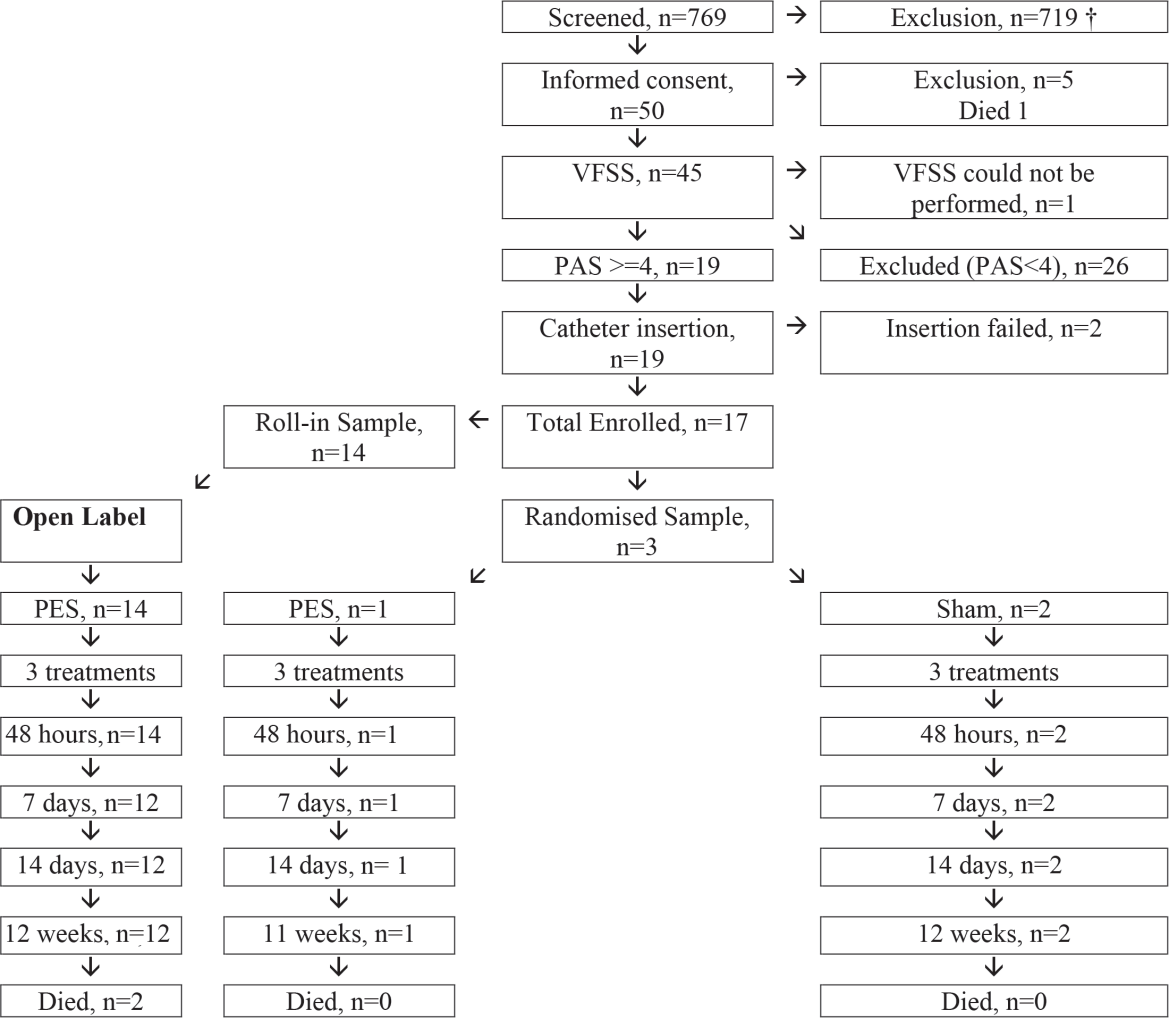
CONSORT diagram showing flow through trial. † Reasons for exclusion (1,005, potential participants may have had > 2 exclusion): 164 – outside of 7–28 day recruitment window; 113 – brainstem stroke; 94 – other conditions preventing participation and/or follow-up; 81 – TBI or SAH; 58 – presence of tracheostomy.

Due to a low recruitment rate, the trial was stopped after recruitment of 17 patients from 11 sites (USA 6, Europe 5) with 14 receiving PES in the roll-in phase only, 1 randomized to PES, and 2 randomized to sham (Fig. 1). A key reason explaining the low recruitment was the frequent exclusion of enrolled participants because the baseline penetration-aspiration scale score, as measured using VFS, was < 4, indicating mild or absent dysphagia. Due to the limited number of participants the statistical plan was modified to present information on those receiving PES (open-label and randomized, *n* = 15) and sham (randomized, *n* = 2); no analyses were performed comparing PES vs sham or in subgroups of participants. Overall, the average age was, median [interquartile range], 65 [62, 74] years with most participants being male (71%) (Table I). 18% of participants had a posterior circulation stroke syndrome. Time from onset to randomization and treatment averaged 11 [9, 15] and 16 [12, 23] days respectively. Six (35%) were tube fed.

**Table I:** Baseline characteristics by diagnostic group in participants where pharyngeal electrical stimulation (PES) catheter insertion was attempted or succeeded

Item	n	All	Roll-in PES	Randomized PES	Randomized sham
*n*	17	17	14	1	2
Age (years)	17	65 [62, 74]	66.5 [63, 74]	61	68 [62, 74]
Sex, male, *n* (%)	17	12 (71)	11 (79)	0 (0)	1 [50)
Region, *n*	17				
North America		9	7	1	1
Europe		8	7	0	1
Medical, *n* (%)					
Atrial fibrillation	17	1 (6)	1 (7)	0 (0)	0 (0)
Alcohol	17	6 (35)	4 (29)	1 (100)	1 (50)
IHD	17	2 (12)	0 (0)	1 (100)	1 (50)
Carotid stenosis	16	0 (0)	0 (0)	0 (0)	0 (0)
Heart failure	17	0 (0)	0 (0)	0 (0)	0 (0)
COPD	17	1 (6)	0 (0)	0 (0)	1 (50)
Renal disease	17	1 (6)	1 (7)	0 (0)	0 (0)
Renal failure	17	1 (6)	1 (7)	0 (0)	0 (0)
Diabetes	16	2 (12.5)	1 (8)	0 (0)	1 (50)
Hyperlipidaemia	16	3 (19)	2 (14)	1 (100)	0 (0)
Hypertension	17	8 (47)	6 (43)	1 (100)	1 (50)
PAD	17	1 (6)	1 (7)	0 (0)	0 (0)
Smoker, current	17	5 (29)	3 (21)	0 (0)	2 (100)
VTE	17	0 (0)	0 (0)	0 (0)	0 (0)
OTR, days, median [IQR range]	17	11 [9, 15]	11 [8, 16]	9	13 [12, 13]
OTT, days, median [IQR range]	17	16 [12, 23]	16 [10, 23]	15	17 [16, 18]
Weight, kg, median [IQR range]	17	80 [68, 84]	80.5 [68, 84]	91	64.5 [53, 76]
Height, m, median [IQR range]	17	1.80 [1.70, 1.83]	1.80 [1.75, 1.85]	1.63	1.68 [1,55, 1.82]
BMI, kg/m^–2^, median [IQR range]	17	25 [23, 29]	25 [23, 29]	34	22.5 [22, 23]
Feeding status, *n* (%)	17				
Oral, modified		10 (59)	8 (57)	1 (100)	1 (50)
NGT/NJT		5 (29)	4 (29)	0 (0)	1 (50)
PEG/RIG		1 (6)	1 (7)	0 (0)	0 (0)
NBM		1 (6)	1 (7)	0 (0)	0 (0)
PEG/NGT, *n* (%)	17	6 (35)	5 (36)	0 (0)	1 (50)
IDDSI, median [IQR range]					
Drinks (/5)	17	1.5 [1.5, 5]	1.5 [1.5, 5]	1.5 [–, –]	4 [3, 5]
Food (/7)	17	2 [2, 4.5]	3.3 [2, 4.5]	7 [–, –]	2 [2, 2]
Score (/8)	17	3 [0, 4]	3 [0, 4]	6.5 [–, –]	0.4 [0, 0.7]
NIHSS (/42), *n* (%)	17	8 (3, 8)	7 (3, 13)	10	7 (1, 13)
OCSP, *n* (%)	15				
TACS		2 (12)	2 (14)	0 (0)	0 (0)
PACS		8 (47)	6 (43)	1 (100)	1 (50)
LACS		1 (6)	1 (7)	0 (0)	0 (0)
POCS		3 (18)	3 (21)	0 (0)	0 (0)
Stroke, ischaemic, *n* (%)	17	15 (88)	13 (93)	9 (0)	2 (100)
Lesion location, *n* (%)	17				
Right		10 (59)	7 (50)	1 (100)	2 (100)
Left		5 (29)	5 (36)	0 (0)	0 (0)
Bilateral		2 (12)	2 (14)	0 (0)	0 (0)
Stroke location^a^, *n* (%)	17				
Cortical		12 (71)	10 (71)	1 (100)	1 (50)
Sub-cortical		9 (53)	7 (50)	1 (100)	1 (50)
Posterior		1 (6)	1 (7)	0 (0)	0 (0)

a Non-exclusive.

IQR: interquartile range; BMI: body mass index; COPD: chronic obstructive pulmonary disease; IDDSI: international dysphagia diet standardization initiative; IHD: ischaemic heart disease; NBM: nil by mouth; NGT: nasogastric tube; NIHSS: National Institute Health Stroke Scale; NJT: nasojejunal tube; OTR: onset to randomization; OTT: onset to treatment; PAD; peripheral arterial disease; PEG: percutaneous gastrostomy tube; RIG: radiographically inserted gastrostomy tube; VTE: venous thromboembolism; IDDSI: International Dysphagia Diet Standardization Initiative (30) derived from DSRS (Ref. 31, Table 6). If nil by mouth, food and drinks scored extended to 2 and 5 respectively. IDDSI score calculated from IDDSI food and drinks (Ref. 45, Fig. 2). All IDDSI scores interpolated where necessary.

In semi-quantitative analyses, MBSImP metrics showed that both groups exhibited physiological impairments at baseline, although soft palate elevation and pharyngeal stripping wave were minimally impaired (Table II). Patients in the sham group had slightly more impaired lip closure and epiglottic inversion whilst patients in the PES group had slightly more impaired lingual motion. Overall at baseline, the sham group had more impaired (higher) oral and pharyngeal component scores than the PES group, with a greater difference in the pharyngeal component scores. MBSImP scores at 48 h following intervention are presented in Table II for comparison.

**Table II:** MBSImP mean overall impression scores

MBSImP components	Baseline		48 h after intervention	
	PES	Sham	PES	SHAM
Oral				
1. Lip closure	1.6	3.0	2.5	4.0
4. Bolus transport/lingual motion	1.8	1.0	2.0	1.5
5. Oral residue	2.1	2.0	2.0	2.0
6. Initiation of pharyngeal swallow	2.7	3.0	3.0	2.5
Oral total sum score	8.1	9.0	9.2	10.0
Pharyngeal				
7. Soft palate elevation	0.1	0.5	1.0	0.0
8. Laryngeal elevation	1.0	1.0	1.0	1.0
9. Anterior hyoid excursion	1.1	1.0	1.0	1.0
10. Epiglottic movement	1.1	2.0	1.0	1.0
11. Laryngeal vestibular closure	1.0	1.0	1.0	1.0
12. Pharyngeal stripping wave	0.6	0.5	1.0	0.5
14. PES opening	1.1	1.5	2.0	1.0
15. Tongue base retraction	2.1	2.5	2.0	2.5
16. Pharyngeal residue	2.3	2.5	0.0	2.5
Pharyngeal total sum score	10.4	12.5	10.0	10.5

Not assessed:

No solids given: components 2, oral (tongue control during bolus hold); and 3, pharyngeal (mastication).

Anteroposterior view imaging not done: components 13, contraction; and 17, oesophageal clearance.

PES stimulation was delivered at 26.6 mA (mean over 3 days), 1 mA below the calculated stimulation level as based on threshold (12.9 mA) and tolerability (32.1 mA) levels (Table III).

**Table III:** Threshold, tolerability, and stimulation treatment currents (mA)

Stimulation, mA	Mean (SD) (over 3 days)
Participants	15
Threshold	12.9 (6.4)
Tolerability	32.1 (7.6)
Calculated	27.6 (6.6)
Stimulation	26.6 (6.6)

SD: standard deviation.

### Revised primary outcome

Participants had significant dysphagia at baseline (median DSRS 8 of total score 12) (Table IV); qualitatively, participants receiving PES had milder dysphagia than those receiving sham: 6 [5, 12] vs 11 [10, 12] although this did not differ statistically (2p = 0.28). DSRS fell significantly with PES from 6 to 2 over 83 days, –4 [–8, –3] (2p = 0.002), with incremental reductions present at days 7 and 14. Improvement was seen with PES over follow-up for 2 of the DSRS sub-categories, diet and supervision, with reductions of 2 and 1 point respectively. The baseline DSRS fluid score in the PES group was low at start (1 out of 4) thereby limiting any ability to see improvement (Table IV). The 2 participants in the sham group had maximal DSRS scores and sub-scores. The effect of PES on DSRS in subgroups was not studied due to the small sample size.

**Table IV:** Primary and secondary swallowing, penetration and aspiration outcomes by treatment with PES v sham

Item	All	PES	Sham
	med [IQR], *n*	med [IQR], *n*	med [IQR], *n*
Total DSRS [/12]			
Day 0	8 [5, 12], 17	6 [5, 12], 15	11 [10, 12], 2
Day 7	8 [3, 12], 15	5 [3, 10], 13	12 [12, 12], 2
Day 14	5 [2, 10], 15	5 [2, 8], 13	12 [12, 12], 2
Day 83	4 [1, 12], 15	2 [0, 4], 13	12 [12, 12], 2
Difference in median	–	–4 [–8, –3] 2p=0.002	–
DSRS diet [/4]			
Day 0	4 [3, 4], 17	3 [3, 4], 15	4 [4, 4], 2
Day 7	3 [2, 4], 15	3 [2, 4], 13	4 [4, 4], 2
Day 14	3 [1, 4], 15	2 [1, 3], 13	4 [4, 4], 2
Day 83	1 [0, 4], 15	1 [0, 2], 13	4 [4, 4], 2
Difference in median	–	–2 [–3, –2] 2p=0.003	–
DSRS fluid [/4]			
Day 0	1 [1, 4], 17	1 [1, 4], 15	3 [2, 4], 2
Day 7	, 15	1 [0, 2], 13	4 [4, 4], 2
Day 14	1 [0, 4], 15	1 [0, 2], 13	4 [4, 4], 2
Day 83	1 [0, 2], 15	0 [0, 1], 13	4 [4, 4], 2
Difference in median	–	–1 [–2, 0] 2p=0.25	–
DSRS supervision [/4]			
Day 0	4 [1, 4], 17	2 [1, 4], 15	4 [4, 4], 2
Day 7	3 [0, 4], 15	2 [0, 4], 13	4 [4, 4], 2
Day 14	1 [0, 4], 15	1 [0, 3], 13	4 [4, 4], 2
Day 83	0 [0, 4], 15	0 [0, 1], 13	4 [4, 4], 2
Difference in median	–	–1 [–4, –1] 2p=0.004	–
FOIS [/7]			
Day 0	3 [1, 5], 17	4 [1, 5], 15	1.5 [1, 2], 2
Day 7	3 [2, 5], 15	5 [2, 5], 13	1.5 [1, 2], 2
Day 14	5 [2, 6], 15	5 [2, 6], 13	1.5 [1, 2], 2
Day 83	5 [1, 7], 15	5 [5, 7], 13	1 [1, 1], 2
Difference in median	–	2 [0, 3] 2p=0.010	–
PAS, thin [/8]			
Day 0	5 [4, 7], 17	5 [4, 7], 15	7 [5, 8], 2
Day 5	4 [2, 7], 17	4 [2, 7], 15	5 [3, 8], 2
Median difference	–	–0.5 [–1.5, 0.5], 2p=0.14	–
PAS, nectar [/8]			
Day 0	5 [2, 6], 15	3 [2, 6], 13	7 [6, 8], 2
Day 5	3 [2, 4], 16	2 [1, 3], 14	5 [3, 6], 2
Difference in median	–	–1.5 [–3, 0.5], 2p=0.25	–

Data are median [interquartile range] and number of participants (med [IQR], *n*), and difference in median [95% confidence intervals]. Comparisons using Mann–Whitney *U* test for difference in medians and 1-sample exact permutation test for median differences. Participants who died are excluded.

PES: pharyngeal electrical stimulation; DSRS: dysphagia severity rating scale; FOIS: functional oral intake scale; PAS: penetration-aspiration scale.

DSRS scored as sum of:

Fluids: 0, Normal/thin fluids (IDDSI level 0); 1, Syrup/slightly–mildly thick consistency (IDDSI level 1/2); 2, Custard/moderately thick consistency (IDDSI level 3); 3, Pudding/extremely thick consistency (IDDSI level 4); 4 No oral fluids.

Diet: 0, Normal diet (IDDSI level 7); 1, Selected textures/easy chew (IDDSI 7); 2, Soft/moist bite-sized (IDDSI level 6); 3, Puree (IDDSI level 4) or minced & moist (IDSSI level 5); 4, Non-oral feeding.

Supervision: 0, Eating independently; 1, Eating with supervision; 2, Feeding by third party (untrained); 3, Therapeutic feeding (trained); 4, N oral feeding.

### Secondary outcomes

PES was associated with a significant increase (improvement) in FOIS of 2 points (see Table IV). Other secondary outcome measures in the PES group, including instrumentally assessed PAS scores for both thin (IDDSI 0) and mildly thick (IDDSI 2) liquid consistencies (Table IV), NIHSS, mRS, Barthel Index, and EQ-5D-5L tended to improve over 83 days of follow-up but none of the changes were statistically significant (Table V). No qualitative change in EQ-VAS was seen. Ten (67%) participants treated with PES were discharged home. Blood pressure and heart rate did not show any systematic changes during or following PES and did not go outside the normal range (Table VI); no clinical symptoms of hyper/hypotension were noted. ECG strips recorded no tachy/bradycardia during or shortly after PES.

**Table V:** Clinical outcomes by treatment with pharyngeal electrical stimulation (PES) vs sham

	All *n* = 17	PES *n* = 15	Sham *n* = 2
NIHSS (/42)			
Day 0	17, 8 [3, 13]	15, 8 [3, 13]	2, 7 [1, 13]
Day 14	15, 7 [2, 13]	13, 7 [2, 10]	2, 8 [2, 13]
Day 83	14, 3 [1, 8]	12, 3 [1, 7]	2, 7 [0, 13]
Difference in median	–	–4 [–9, 0], 2p=0.056	–
mRS (/5)			
Day 0	17, 4 [3, 5]	15, 4 [3, 5]	2, 4 [3, 4]
Day 14	15, 4 [3, 4]	13, 4 [3, 4]	2, 4 [3, 4]
Day 83	15, 4 [3, 4]	13, 3 [3, 4]	2, 4 [4, 4]
Difference in median	–	–1 [–2, –1], 2p=0.098	–
Barthel index (/100)			
Day 0	16, 25 [8, 55]	14, 25 [5, 55]	2, 48 [10, 85]
Day 14	15, 35 [0, 55]	13, 35 [0, 50]	2, 60 [50, 70]
Day 83	13, 60 [30, 80]	11, 65 [15, 85]	2, 40 [35, 45]
Difference in median	–	23 [–5, 55], 2p=0.074	–
EQ-5D-5L utility (/1)			
Day 0	16, 0.43 [0.18, 0.61]	14, 0.43 [0.18, 0.62]	2, 0.36 [0.18, 0.54]
Day 83	14, 0.67 [0.38, 0.77]	13, 0.68 [0.40, 0.77]	1, 0.26 [–]
Difference in median	–	0.16 [–0.06, 0.36], 2p=0.089	–
EQ-VAS (/100)			
Day 0	14, 50 [20, 60]	12, 50 [30, 55]	2,40 [20, 60]
Day 83	14, 50 [40, 60]	13, 50 [40, 60]	1, 40 [–]
Difference in median	–	3 [–25, 20], 2p=0.60	–
Discharge destination			
Length of stay/death (days)	14, 46 [27, 75]	13, 45 [27, 75]	1, 53 [–]
Death	17, 2 (11.8)	15, 2 (13.3)	2, 0 (0.0)
Hospital	2 (11.8)	1 (6.7)	1 (50.0)
Inpatient rehabilitation	2 (11.8)	2 (13.3)	0 (0.0)
Care home	1 (5.9)	0 (0.0)	1 (50.0)
Home (with care)	7 (41.2)	7 (46.7)	0 (0.0)
Home (independent)	3 (17.6)	3 (20.0)	0 (0.0)

Data are number of participants, median [interquartile range], and difference in median [95% confidence intervals]. Comparisons using Mann–Whitney *U* test. Participants who died are excluded.

**Table VI:** Blood pressure (mmHg) and heart rate (beats per minute) immediately prior to and immediately after each daily pharyngeal electrical stimulation (PES) treatment

Haemodynamic measures	All *n* = 17	PES *n* = 15	Sham *n* = 2
Blood pressure, systolic			
Treatment 1			
Before	117 [109, 125]	117 [109, 125]	125 [99, 151]
After	115 [112, 132]	115 [112, 132]	120 [96, 144]
Median difference	–	–3 [–10, 2] 2p=0.21	–
Treatment 2			
Before	120 [107, 132]	120 [107, 132]	119 [88, 149]
After	129 [116, 142]	129 [116, 142]	122 [95, 149]
Median difference	–	7 [2, 20] 2p=0.003	–
Treatment 3			
Before	120 [116, 134]	120 [116, 138]	116 [98, 134]
After	118 [113, 137]	118 [113, 137]	122 [96, 147]
Median difference	–	–1 [–8, 9] 2p=0.62	–
Blood pressure, diastolic			
Treatment 1, before			
Before	66 [58, 76]	67 [57, 77]	62 [58, 66]
After	65 [60, 72]	70 [57, 80]	61 [60, 61]
Median difference	–	1 [0, 4] 2p=0.19	–
Treatment 2			
Before	62 [57, 80]	62 [57, 80]	73 [57, 88]
After	72 [60, 82]	72 [60, 85]	66 [54, 78]
Median difference	–	3 [–1, 15] 2p=0.30	–
Treatment 3			
Before	68 [62, 82]	68 [62, 83]	68 [59, 76]
After	66 [62, 75]	66 [59, 81]	68 [65, 71]
Median difference	–	1 [–4, 3] 2p=0.45	–
Heart rate			
Treatment 1			
Before	75 [68, 88]	77 [67, 89]	71 [68, 73]
After	79 [71, 88]	80 [70, 88]	78 [77, 78]
Median difference	–	0 [0, 2] 2p=0.66	–
Treatment 2			
Before	78 [74, 89]	77 [72, 89]	85 [78, 91
After	79 [75, 85]	79 [71, 85]	79 [75, 83]
Median difference	–	1 [–4, 7] 2p=0.50	–
Treatment 3			
Before	80 [77, 86]	80 [76, 95]	83 [80, 86]
After	83 [75, 89]	83 [75, 89]	84 [81, 87]
Median difference	–	0 [–3, 4] 2p=0.79	–

Data are median [interquartile range]. Median difference [95% confidence intervals] calculated using 1-sample exact permutation test.

In semi-quantitative MBSImP analyses, patients treated with PES showed greater improvement in pharyngeal total sum scores (33.3%) compared with oral total sum scores (20.0%), with most improvements observed in components: 10, epiglottic inversion (13.3%); 14, pharyngoesophageal segment opening (20.0%); 15, tongue base retraction (13.3%); and 16, pharyngeal residue (20.0%). Improvements were also observed in components: 4, bolus transport/lingual motion (6.7%); 6, initiation of pharyngeal swallow (6.7%); 7, soft palate elevation (7.1%); and 9, anterior hyoid excursion (6.7%).

### Adverse events

There were 23 adverse events (AEs) in 7 participants (41.1%), 10 serious adverse events (SAEs) in 4 participants (23.5%), and 2 adverse device events (ADEs) in 2 participants (11.8%); overall, 13 of 17 (76.5%) participants experienced an event. One ADE comprising otalgia was considered definitely related and 1 event of cough during catheter placement was considered possibly related. Both ADEs were classified as mild in severity and resolved within 1 and 2 days respectively so that neither led to withdrawal of the participants from the study.

The SAEs all occurred in the PES group and included anaemia (1), gastrointestinal bleeding (2), acute respiratory failure (1), dyspnoea (1), aspiration pneumonia (2), pneumothorax (1), and pulmonary embolism (1). An independent medical monitor determined that none of the SAEs were related to study procedure or to the investigational device. One participant suffered a seizure shortly after providing informed consent, developed an aspiration pneumonia and died prior to VFS or catheter insertion; these SAEs were unrelated to study procedures. Nasopharyngeal clinical examination in all participants at days 7 and 14 revealed no trauma from the catheter.

## DISCUSSION

Our study has shown that the use of PES is associated with a reduction in swallowing impairment as measured by the DSRS and FOIS in patients with early PSD. The reductions in DSRS from 1 to 4 points at days 7, 14, and 83 all equalled or exceeded the minimum clinically important difference of 1 (31). The use of PES was safe, with no off-target clinical effects during active treatment and only 2 mild non-serious adverse reactions during catheter placement that quickly resolved. Use of PES had no impact on cardiac rhythm or function. This study adds to the existing literature supporting the potential impact and safety of PES in the treatment of PSD.

Dysphagia after stroke results from damage to the swallowing network, which includes cortical structures (36). Swallowing muscles have discrete somatotopic representation in the bilateral motor and premotor cortex, with consistent lateralization that is independent of handedness (37). Post-stroke dysphagia is often caused by injury to the hemisphere dominant for swallowing, and recovery of swallow function is associated with expansion of the smaller surviving representation in the uninjured (non-dominant) hemisphere. In contrast, the non-dominant swallow representation remains unchanged if dysphagia persists (38, 39). PES has been shown to increase cortical excitability in cortical regions representing the pharynx via stimulation of pharyngeal sensory pathways (40–42). Furthermore, PES can increase salivary levels of substance P, a neuropeptide known to enhance the swallow response (43). In healthy volunteer studies, PES reversed the effects of a virtual lesion in the pharyngeal cortex following inhibitory repetitive transcranial magnetic stimulation (17) and increased brain activations after lidocaine-induced pharyngeal hypoesthesia (44). An individual patient data meta-analysis of pilot clinical studies showed that PES was associated with improvements in PAS and DSRS (21).

The findings presented here are noteworthy in that they suggest PES could be effective in patients with less severe PSD, i.e., DSRS = 6, FOIS = 4. The PHADER single-arm observational cohort study included 255 patients with neurogenic dysphagia, all of whom were treated with PES (15); patients had more severe PSD (DSRS 10.9, FOIS = 1.7) and PES treatment was associated with a reduction in swallow impairment. Taking PhEED and PHADER together, the observations suggest that PES might be effective in both moderate and severe PSD.

The largest randomized study of PES was the STEPS trial (14), for which 123 participants with PSD and not requiring any mechanical ventilation or tracheostomy were recruited; this study was neutral but suffered from low PES stimulation levels averaging 14.7 mA, just 5.3 mA above threshold. These were lower than those achieved here with a mean stimulation of 26.6 mA, i.e., 13.7 mA above threshold. Participants randomized to sham in STEPS also received some stimulation as part of determining threshold and tolerability levels and these may have had potential neuromodulatory impact (14). By contrast, the PHAST-TRAC randomized trial focused on improving FEES-based swallowing measures to facilitate decannulation in 69 stroke patients with tracheostomy. PES was associated with an increase in decannulation within 24–72 h after treatment as compared with sham (49% vs 9%) (16). These studies, including PHADER, have shown that PES has an excellent safety profile (11, 15, 16).

### Limitations

The primary limitation of this study was recruitment difficulty, thereby leading to a small sample size, and the inability to provide valid comparison between participants randomized to treatment vs sham. After meeting entry criteria, many subjects were excluded because screening with VFS demonstrated that swallow dysfunction was either mild (PAS < 4) or absent, allowing advancement to oral diet and thin liquids by mouth. Recruiting subjects earlier post-stroke in the acute phase, rather than later in the rehabilitation phase, might have improved recruitment rate and completion of the study as planned. The small sample sized makes it difficult to exclude the possibility of spontaneous recovery of swallow function early post-stroke. Because of low recruitment, the data presented here represent a case series rather than a clinical trial, and may be interpreted in the context of previous research, as well as to inform the interpretation of future studies. As such, the statistical analysis presented is exploratory in nature. However, because the study was designed as a clinical trial, all study procedures were standardized as were the outcome measures supporting the accuracy of the findings.

### Conclusion

PES was associated with improvements in swallowing impairment assessed using the DSRS. Our data support the findings of previous studies. Further large randomized clinical trials evaluating the efficacy of PES vs no PES for PSD recovery are warranted. One such study is the ongoing UK government-funded pharyngeal electrical stimulation for acute stroke dysphagia trial (PhEAST), which is assessing the safety and effectiveness of PES in 800 tube-feeding-dependent patients with PSD randomized between 2 and 31 days after stroke (https://www.isrctn.com/ISRCTN98886991).
